# Relation of Alcoholic Indulgence to Insanity

**Published:** 1897-03

**Authors:** 


### Relation of Alcoholic Indulgence to Insanity.

1. Alcoholic excesses produce insanity.

2. They are directly the cause of at least 10 to 12 percent, and probably of a somewhat larger percentage. Indirectly they are among the casual factors of a very large proportion of cases that can not be directly attributed to alcohol.

3. Moderate drinking is a very indefinite term, and this fact alone makes it impossible to utilize satisfactorily any statistics as to its effect in producing mental disease. There is, however, no reason to believe that moderate indulgence in alcohol is specially conducive to mental health in the average individual, and there is, on the other hand, a certain amount of physiological *a priori* presumption to the contrary. For the victim of hereditary taint or the neurotic it is undoubtedly often disastrous in its effects in this direction.—*American Journal of Insanity.*

